# 

**Published:** 1895-07

**Authors:** 


### CONTENTS.

ORIGINAL ARTICLES—

Appendicitis: A Brief Review of My Personal Experience. By Floyd Wilcox McRae, M. D., Atlanta .......... 343

Ten Minutes in Medical Electricity. By Geo. S. Hull, Ph. G., M. D., Chambersburg, Pa .......... 350

A Case of Insanity Cured by Removal of a Fibroid Tumor of the Ovary. By Emory Lanphear, M. D., Ph. D., St. Louis, Mo ..... 355

Complications Following Spinal Caries, With Report of Cases. By Samuel Milliken, M. D., New York .......... 358

Clinical Lecture on Hernia. By W. B. De Garmo, M. D .......... 361

New York Letter .......... 365

SOCIETY REPORTS—

Philadelphia Academy of Surgery .......... 368

Report of a Case of Osteo-Lipo-Chondroma of the Upper End of the Humerus. Resection of Humerus. Exhibition of Patient. By Thos. R. Neilson, M. D .......... 371
Remarks Upon Trephining the Cranium. By John Ashhurst, Jr., M. D. .......... 373

SELECTIONS AND ABSTRACTS—

Spermatorrhea with Nocturnal Pollutions .......... 378
New Method of Inducing General Narcosis .......... 379
A New Method of Making Palatable and Digestive Milk .......... 379

EDITORIAL—

Announcement .......... 380
The Gynæcological Fad .......... 381
Notes .......... 382
Book Reviews .......... 385
Special Notes .......... 388

